# BK channel clustering is required for normal behavioral alcohol sensitivity in *C*. *elegans*

**DOI:** 10.1038/s41598-019-46615-9

**Published:** 2019-07-15

**Authors:** Kelly H. Oh, Hongkyun Kim

**Affiliations:** 1 Center for Cancer Cell Biology, Immunology, and Infection, Department of Cell Biology & Anatomy, Chicago Medical School, North Chicago, Illinois 60064 USA; 2 School of Graduate and Postdoctoral Studies, Rosalind Franklin University, North Chicago, Illinois 60064 USA

**Keywords:** Molecular neuroscience, Genetics of the nervous system

## Abstract

The large conductance, calcium- and voltage-activated potassium channel, known as the BK channel, is one of the central proteins that mediate alcohol intoxication and tolerance across species. Although ethanol targets BK channels through direct interaction, how ethanol-mediated BK channel activation causes behavioral intoxication is poorly understood. In. *C*. *elegans*, loss of function in SLO-1, the BK channel ortholog, confers profound ethanol resistance in movement and egg-laying behaviors. Here, we show that depletion of SLO-1 channels clustered at the active zones with no change in the overall channel expression level results in locomotory resistance to the intoxicating effect of ethanol, equivalent to that of *slo-1* loss-of-function mutants. Likewise, depletion of clustered SLO-1 channels in the sarcolemma and neurons leads to ethanol-resistant egg-laying behavior. By contrast, reduction in the overall SLO-1 channel level by over 70% causes only moderate ethanol resistance in movement, and minimal, if any, resistance in egg laying. Our findings strongly suggest that behavioral ethanol sensitivity is conferred by local, but not global, depression of excitability via clustered BK channels. Given that clustered BK channels are functionally coupled to, and localize near, calcium channels, ethanol may mediate its behavioral effects by targeting BK channels and their coupled calcium channels.

## Introduction

BK channels have emerged as a major mediator of alcohol response across species from *C*. *elegans* to humans^1–3^. In *C*. *elegans* genetic screens designed to identify genes that mediate the intoxicating effects of ethanol, *slo-1* mutants were most frequently identified and showed profound resistance^4^. Moreover, ethanol-mediated BK channel activation is commonly observed in mice, rats, and humans, indicating that the effect of ethanol on BK channels is an evolutionarily conserved phenomenon^5^. Recent studies of *C*. *elegans*, mouse, and human BK channels have demonstrated that single amino acid mutations in the cytoplasmic C-terminal domain abolish ethanol-mediated BK channel activation without altering overall channel properties^6,7^, suggesting that ethanol activates BK channels through direct interaction with these amino acids. Importantly, the fact that these potential ethanol-binding sites are positioned away from known calcium binding sites suggests that ethanol may act on BK channels independently of calcium binding, although it is difficult to completely exclude the possibility that they may affect calcium binding indirectly through an allosteric gating mechanism. However, it is worthwhile to note that the consequence of the interaction between BK channels and ethanol is not straightforward in mammals^2,3^. While ethanol activates BK channels at calcium ion concentration below 20 μM, ethanol inhibits BK channels as calcium ion concentrations further increase^8^. Furthermore, β subunits of mammalian BK channels influence the calcium sensitivity of the channels and thus shift the range of calcium concentrations at which ethanol results in potentiation^9^. Given that the range of intracellular calcium concentrations is highly variable among different subcellular compartments, it is an open question whether ethanol uniformly targets all of the BK channels present in the plasma membrane or prefers certain types of BK channels that mediate the behavioral effects of ethanol.

Studies on SLO-1 in *C*. *elegans* provided insights into its localization and function in muscles and neurons^10,11^. CTN-1, an α-catulin ortholog that shares homology with α-catenin and vinculin^12^, clusters SLO-1 channels at either muscle excitation sites or presynaptic terminals by direct physical interaction^13–16^, and ERG-28 (a homolog of ergosterol biosynthetic protein 28), an endoplasmic reticulum (ER) membrane protein, promotes the trafficking of SLO-1 channels from the ER^17^. Consistent with these cell biological data, *ctn-1* and *erg-28* mutants were initially isolated as suppressors of *slo-1* (*gf*, *gain-of-function*) phenotypes and were found to have deficits in evoked synaptic responses in electrophysiological recordings at the neuromuscular junction^13,15^. Despite their common involvement in the SLO-1-mediated synaptic response, ERG-28, but not CTN-1, is required for the inhibitory function of SLO-1 channels in calcium signaling during the establishment of the left-right asymmetry of AWC (Amphid Wing C cell) olfactory neurons^13,17,18^. This raised the question of how these two different genes distinctively contribute to SLO-1 function.

In this study, we used *C*. *elegans* to investigate how alcohol intoxication at the behavioral level is mediated via SLO-1 channels. Using different mutants that affect either SLO-1 channel clustering or density, we discovered that clustered, but not diffuse, SLO-1 channels at presynaptic terminals and presumed muscle excitation sites mediate the intoxicating effect of ethanol.

## Results

### *ctn-1* mutants exhibit more prominent ethanol-resistant locomotory behavior than *erg-28* mutants

**Figure 1:**
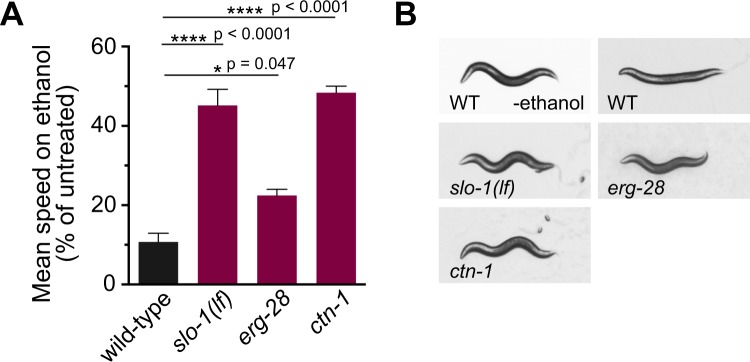
*ctn-1* and *erg-28* mutations confer differential resistance to the intoxicating effect of ethanol. (**A**) The locomotory speeds of wild-type, *slo-1*(*eg142*), *erg-28*(*gk697770*), or *ctn-1*(*eg1167*) mutant animals were measured in the presence of ethanol and their values were divided by the average speed of untreated animals. Error bars represent S.E.M. One-way ANOVA with Tukey’s post-hoc analysis (wild-type vs. *erg-28*: p = 0.0473, wild-type vs. *ctn-1*: p < 0.001, wild-type vs. *slo-1*: p < 0.001, *slo-1* vs. *ctn-1*: p = 0.8136). Data points represent three independent trials of 10 animals. See Supplementary Video 1. (**B**) Snapshots of single animals in the presence of an intoxicating dose of ethanol show sinusoidal posture.

The *ctn-1* and *erg-28* genes were previously identified as regulators of SLO-1 in a genetic screen to identify suppressors of sluggish movement of the *slo-1*(*ky399*) gain-of-function mutant^13,17^. While mutations in either gene suppress the sluggish movement of the *slo-1*(*ky399gf*) mutant, only an *erg-28*, but not a *ctn-1*, mutation suppresses the defective asymmetric differentiation of the AWC neuron pair in a *slo-1*(*ky399gf*) mutant^13,17,18^. This prompted us to determine whether mutations in these two genes have differential effects on ethanol intoxication, another well-known *slo-1* function. Although we have recently reported that mutations in *erg-28* confer resistance to the intoxicating effect of ethanol^17^, the effect of *ctn-1* mutation on ethanol-mediated behavior has not been investigated. As previously reported, at an exogenous concentration that causes complete intoxication of the wild-type animals, *slo-1*(*lf*, loss-of-function) mutants exhibited a strongly ethanol-resistant locomotory phenotype^4^, and *erg-28*(*gk697770*) mutants exhibited a moderately ethanol-resistant locomotory phenotype (Fig. 1, Supplementary Video 1). We found that *ctn-1*(*eg1167*) mutants showed as much resistance as *slo-1*(*lf*) mutants (Fig. 1, Supplementary Video 1). The average speeds of these tested animals in the absence of ethanol did not differ by genotype (wild-type: 191 ± 12.3, *erg-28:* 197 ± 11.8, *ctn-1:* 187 ± 8.8, *slo-1:* 197 ± 7.6 μm sec^−1^, mean ± sem). Thus, whereas *erg-28* mutation, but not *ctn-1* mutation, suppresses defective asymmetric differentiation of the AWC neuron pair in the *slo-1*(*ky399gf*) mutant^17^, *ctn-1* mutation is more effective than *erg-28* mutation in abolishing the function of SLO-1 in ethanol intoxication.

### CTN-1 is essential for SLO-1 channel clustering, while ERG-28 is necessary to maintain the levels of SLO-1 channels

**Figure 2:**
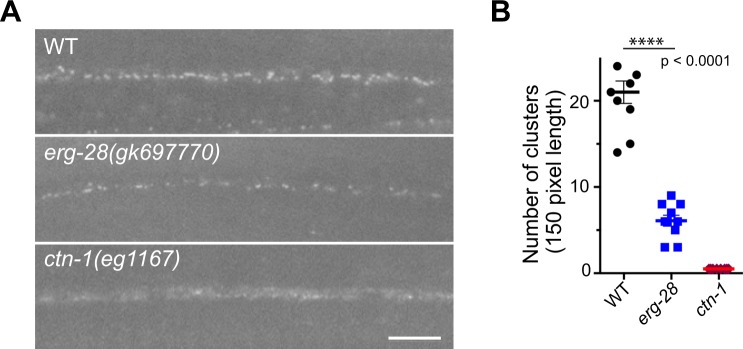
Mutation of the *ctn-1* gene depletes clustered SLO-1 channels in neurons, whereas mutation of the *erg-28* gene reduces the number of clustered SLO-1 channels. (**A**) SLO-1 localization pattern in the dorsal cord of wild-type, *erg-28*(*gk697770*), and *ctn-1*(*eg1167*) mutant animals. The *slo-1*(*cim105[slo-1::GFP]*) strain, which was generated using genome-editing^17^, was used in the analysis. Scale bar, 5 μm. (**B**) The number of SLO-1 puncta representing clustered SLO-1 channels in the dorsal cord. n = 10, Sample numbers represent independent biological replicates.; F(9, 9) = 4.173, p = 0.0447, ANOVA, t(10.23) = 18, p < 0.0001, unpaired two-tailed t-test. *ctn-1* mutants were excluded from the test, since we could not detect stable puncta.

**Figure 3:**
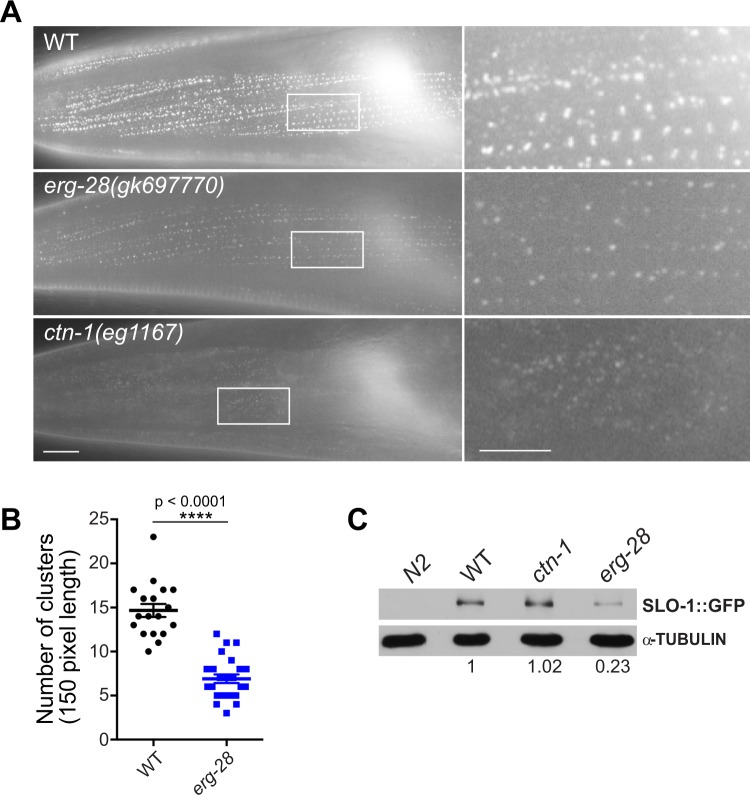
Mutation of the *ctn-1* gene depletes clustered SLO-1 channels in muscle without affecting the overall SLO-1 levels. (**A**,**B**) Representative SLO-1 localization patterns in the sarcolemma of wild-type, *erg-28*(*gk697770*), and *ctn-1*(*eg1167*) mutant animals. The right panels are zoomed-in images of the boxed areas of the left panel images. Note that *ctn-1* mutants exhibit low-level fluorescent SLO-1 signals that fail to form large high-intensity clusters. Scale bar: 5 μm. Comparison of head muscle SLO-1 channel clusters between wild-type and *erg-28*(*gk697770*) animals: F(17, 23) = 1.683, p = 0.2429, ANOVA, t(9.140) = 40, p < 0.0001, unpaired two-tailed t-test. (**C**) Western blot analysis shows that the total SLO-1::GFP ratio is not reduced in *ctn-1* mutants. SLO-1::GFP intensities in wild-type, *erg-28*(*gk697770*), and *ctn-1*(*eg1167*) were normalized to α-tubulin, and their intensities relative to wild-type are indicated. N2 represents a wild type animal that does not express GFP and serves as a negative control for Western blot with an anti-GFP antibody. The blot was probed first with anti-GFP antibody (anti-rabbit monoclonal) and then with anti-α-tubulin antibody (anti-mouse monoclonal) (Supplementary Fig. 3).

What could be the mechanism for these differential effects of *erg-28* and *ctn-1* mutation? ERG-28 is required for efficient trafficking of SLO-1 to the plasma membrane and *erg-28* mutation leads to a drastic reduction in the number of SLO-1 channels, but SLO-1 puncta are still present at presynaptic terminals. CTN-1 is necessary for SLO-1 to cluster at presynaptic terminals. This led us to hypothesize that the function of SLO-1 in ethanol intoxication is critically dependent on its clustering at presynaptic terminals, while its function in AWC neuron differentiation is not. In a previous study on CTN-1^15^, it was not conclusive whether *ctn-1* mutation affect the SLO-1 protein level in addition to clustering because a transgenic line overexpressing SLO-1 in a select group of motor neurons was used. Hence, we used a genome-edited line expressing GFP-tagged SLO-1 to determine SLO-1 expression levels and localization in the *ctn-1* mutant and compared them with the *erg-28* mutant. This genome-edited line, *cim105[slo-1::GFP]*, is indistinguishable from wild-type animals in locomotive speed, body posture and egg-laying behavior^17^. *ctn-1* mutation disrupts SLO-1 clustering at presynaptic terminals and muscle excitation sites (Figs 2 and 3). In *erg-28*(*gk697770*) mutants, the number of clustered SLO-1 channels was reduced to approximately 25% of the quantity in wild type animals. In *ctn-1*(*eg1167*) mutants, the number of clustered SLO-1 channels in the dorsal nerve cord was reduced to a level that was not reliably quantifiable (Fig. 2). In body wall muscle cells, most SLO-1 channels aligned as distinct punctal structures in the sarcolemma. In *erg-28*(*gk697770*) mutants, the clusters of SLO-1 channels were reduced in number compared to those of wild-type animals. In *ctn-1*(*eg1167*) mutants, numerous low, diffused fluorescent signals were present but they did not form high-intensity clusters similar to those in wild-type animals (Figs 2 and 3). These low-intensity fluorescent signals are not readily detectable with a line-scanning quantification method. To further understand the defect of SLO-1 cluster formation in *ctn-1* mutants, we tracked low-level fluorescent signals using time-lapse imaging. Unlike those of wild-type animals, the low-level fluorescent signals of mutants were highly mobile and occasionally formed slightly intense puncta, which quickly dissipated (Supplementary Video 2, Supplementary Fig. 2). Likewise, highly mobile low-level fluorescent signals were observed in the dorsal cord of *ctn-1* mutant animals (Supplementary Video 2). Weak, mobile SLO-1 signals in *ctn-1* mutants could be the result of either endosome-mediated SLO-1 degradation or diffusion of SLO-1 channels in the plasma membrane due to the lack of anchoring. To distinguish these two possibilities, we measured the amount of SLO-1::GFP using Western blot analysis. The total amount of SLO-1::GFP was reduced in *erg-28*(*gk697770*) mutants by over 70% but was not significantly different in *ctn-1*(*eg1167*) mutants when compared to wild-type animals (Fig. 3). This indicates that CTN-1 is absolutely required for SLO-1 clustering and that its absence causes SLO-1 channels to adopt diffuse, mobile forms without promoting degradation.

The inability of *ctn-1* mutation to suppress the defective AWC neuron differentiation of *slo-1*(*gf*) mutants indicates that SLO-1 function is normal in AWC neurons of *ctn-1* mutant animals. Thus, we examined SLO-1 localization in AWC neurons using transgenic animals expressing SLO-1::GFP under the *odr-1* promoter, which is active in AWC and AWB (amphid wing B cell) neurons. Consistent with the results from the nerve cords, SLO-1::GFP is not clustered but diffused in the axons of the *ctn-1* mutant (Supplementary Fig. 1). This suggests that the overall level of SLO-1, rather than clustering, is critical for its function in controlling asymmetric differentiation of AWC neurons. These results further support the idea that SLO-1 channels in *ctn-1* mutant animals remain in the plasma membrane.

### SLO-1 channels clustered at the presynaptic terminals confer ethanol sensitivity in locomotion

**Figure 4:**
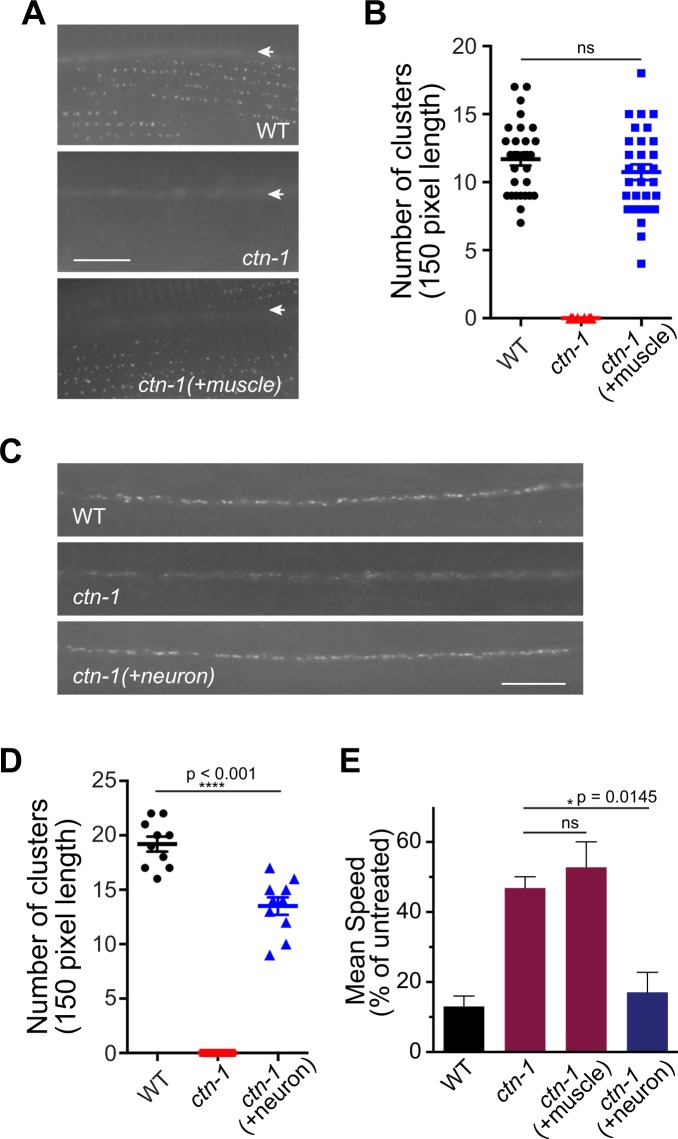
CTN-1-mediated SLO-1 clustering in neurons is critical for alcohol intoxication. (**A**,**B**) Muscle expression of a wild-type *ctn-1* in *ctn-1*(*eg1167*) mutant animals restores SLO-1 clustering in the sarcolemma, but not the neurons in the dorsal cord. The white arrows indicate the dorsal cord. +muscle: *myo-3* promoter-driven expression of *ctn-1*. Scale bar, 10 μm. Comparison of muscle SLO-1 channel clusters between wild-type and muscle-rescued *ctn-1* mutant animals: F(29, 29) = 1.393, p = 0.3770, ANOVA, t(1.296) = 58, p = 0.2002, unpaired two-tailed t-test. (**C**,**D**) Neuronal expression of a wild-type *ctn-1* in *ctn-1*(*eg1167*) mutant animals restores SLO-1 clustering at presynaptic terminals, but not sarcolemma. +neuron: *rgef-1* promoter-driven expression of *ctn-1*. Scale bar,10 μm. Comparison of neuronal SLO-1 channel clusters between wild-type and neuron-rescued *ctn-1* mutant animals: F(9, 9) = 1.406, p = 0.6197, ANOVA, t(5.405) = 18, p < 0.0001, unpaired two-tailed t-test. (**E**) Neuronal, but not muscular, *ctn-1* expression restores normal ethanol-sensitive locomotory behavior. Locomotory speed was measured as in Fig. 1. +muscle: *myo-3* promoter-driven expression of *ctn-1*. +neuron: *H20* pan-neuronal promoter-driven expression of *ctn-1*. Error bars represent S.E.M. One-way ANOVA with Tukey’s post-hoc analysis (*ctn-1* ethanol treated vs. *ctn-1*(+*neuron*) ethanol treated: p = 0.0145, *ctn-1* ethanol treated vs. *ctn-1*(+muscle) ethanol treated: p = 0.8477). Data points represent three independent trials of 10 animals.

A previous study showed that neuronal SLO-1 function is critical for the intoxicating effect of ethanol on locomotory behavior^4^. Expression of the *slo-1* gene in neurons converts the ethanol-resistant locomotory phenotype of *slo-1*(*lf*) mutants to a wild-type-like sensitive phenotype. Therefore, we asked whether the clustering of SLO-1 channels at presynaptic terminals of neurons is essential for mediating alcohol-induced sedation. To answer this question, we first expressed a wild-type copy of *ctn-1* in either muscles or neurons of *ctn-1*(*eg1167*) mutant animals and determined whether SLO-1 channel clusters were restored. Muscular and neuronal expression restored SLO-1 channel clusters in each transgenic line (Fig. 4A–D). Next, we determined whether we could reverse ethanol-resistant locomotory behavior of *ctn-1* mutants in neuronal and muscular transgenic lines (Fig. 4E). We found that neuronal expression, but not muscular expression, of the *ctn-1* gene reversed the ethanol-resistant locomotory behavior of the *ctn-1* mutants (Fig. 4E). Taken together, these results indicate that clustered SLO-1 channels in neurons are responsible for mediating the intoxicating effects of ethanol.

### Ethanol-mediated egg-laying suppression is primarily mediated by clustered SLO-1 channels

**Figure 5:**
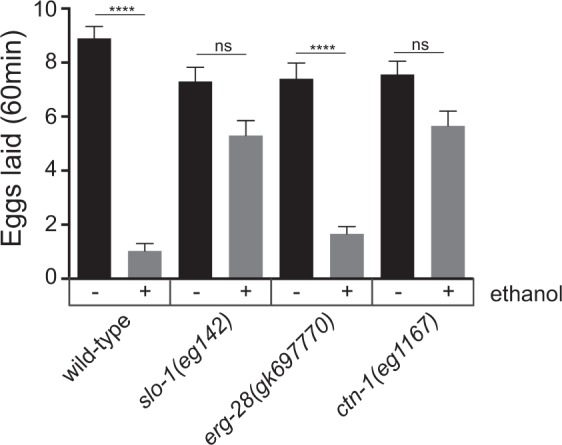
Mutation of the *ctn-1* gene, but not the *erg-28* gene, causes strong ethanol-resistant egg-laying behavior. Egg-laying behavior of wild-type, *slo-1*(*eg142*), *erg-28*(*gk697770*), or *ctn-1*(*eg1167*) mutant animals was quantified by measuring the number of eggs laid over a 60 min period in the presence or absence of ethanol. Egg laying was measured in 10 age-matched animals (30 h post L4) and repeated three times (biological replicates). The data (mean + SEM) are presented as the number of eggs laid by individual animals. One-way ANOVA with Tukey’s post-hoc analysis (wild-type N2 vs. wild-type N2 ethanol treated: ****p < 0.0001, *slo-1* vs. *slo-1* ethanol treated*:* p = 0.1174, *erg-28* vs. *erg-28* ethanol treated: ****p < 0.0001, *ctn-1* vs. *ctn-1* ethanol treated*:* p < 0.1517).

**Figure 6:**
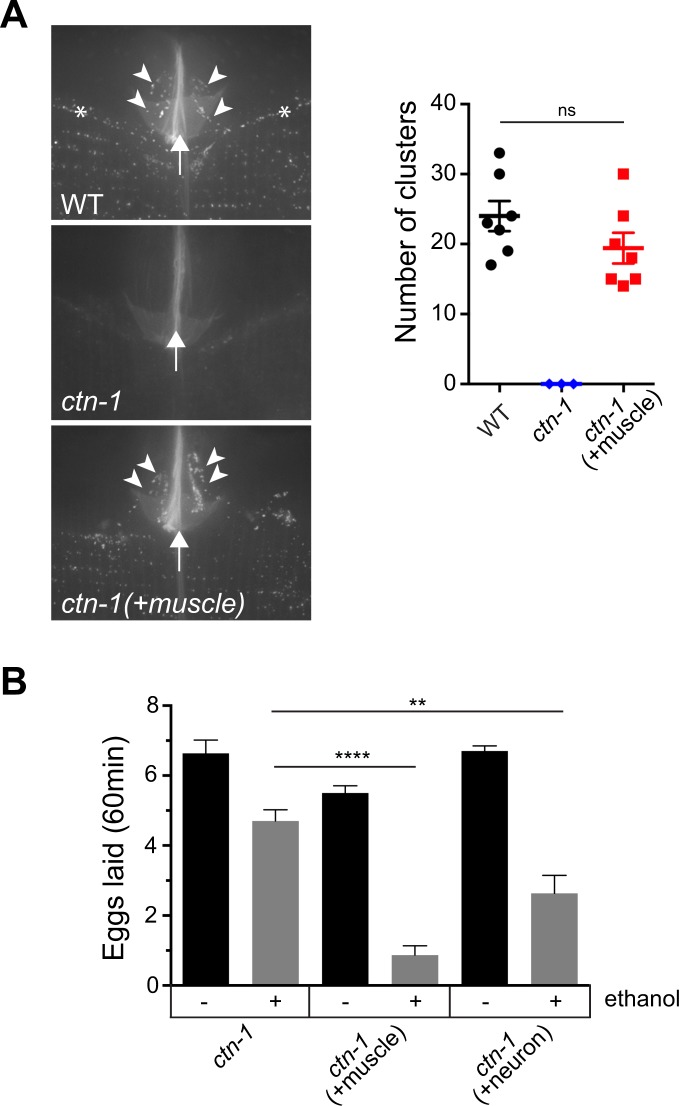
CTN-1-mediated SLO-1 clustering in neurons and egg-laying muscles contributes to ethanol-mediated suppression of egg laying behavior. (**A**) Muscle expression of a wild-type copy of *ctn-1* in *ctn-1*(*eg1167*) mutant animals restores SLO-1 clustering at egg-laying muscles. The images represent a maximum projection of Z-stack image sections. The asterisks, arrows, and arrowheads denote the ventral nerve cord, the vulval slit, and SLO-1 clusters present in egg-laying muscle cells, respectively. The number of SLO-1 channel clusters were quantified from a region of a single muscle cell, which was not overlapped with body wall muscle or the ventral cord. Comparison of vulval muscle SLO-1 channel clusters between wild-type and muscle-rescued *ctn-1* mutant animals: F(6, 6) = 1.039, p = 0.96, ANOVA, t(1.482) = 12, p < 0.1642, unpaired two-tailed t-test. WT: wild-type, +muscle: *myo-3* promoter-driven expression of *ctn-1*. (**B**) Neuronal and muscular SLO-1 channel clustering contributes to ethanol-mediated suppression of egg laying. Egg laying was measured as in Fig. 5. Egg laying was measured in 10 age-matched animals (30 h post L4) and repeated three times (biological replicates). +muscle: *myo-3* promoter-driven expression of *ctn-1*. +neuron: *H20* pan-neuronal promoter-driven expression of *ctn-1*. Error bars represent S.E.M. One-way ANOVA with Tukey’s post-hoc analysis (*ctn-1* ethanol treated vs. *ctn-1*(+*neuron*) ethanol treated: p = 0.0086, *ctn-1* ethanol treated vs. *ctn-1*(+muscle) ethanol treated*:* p < 0.0001).

In addition to locomotory behavior, *slo-1* mutants exhibit robust ethanol-resistant egg-laying behavior. The neural circuits for egg-laying behavior is different from the circuits for locomotory behavior. While locomotory behavior is mediated by striated body wall muscle that receives cholinergic and GABAergic inputs from motor neurons, egg laying is mediated by smooth muscle cells that receive serotonergic and cholinergic inputs from the HSN (Hermaphrodite Specific Neuron) and VC (Ventral Cord) neurons^19^. We examined whether *erg-28*(*gk697770*) and *ctn-1*(*eg1167*) mutants also exhibit ethanol-resistant egg-laying behavior (Fig. 5). In the presence of an ethanol dose that suppresses the egg laying of wild-type animals almost completely, *slo-1* and *ctn-1* mutants laid significantly more eggs over 60 min than wild-type animals (Fig. 5). However, *erg-28* mutants show minimal, if any, egg laying under the same conditions. These results indicate that clustered SLO-1 channels are responsible for ethanol-mediated suppression of egg laying. Next, we asked in which tissue the clustering of SLO-1 channels is responsible for ethanol-mediated suppression of egg laying. Specifically, we investigated whether the ethanol-mediated change in egg laying behavior is mediated through egg-laying muscles or upstream neurons (Fig. 6). Muscle-specific expression of the *ctn-1* gene in *ctn-1*(*eg1167*) mutants restored the SLO-1 clusters in egg laying muscles (Fig. 6A). This restored SLO-1 channel clustering reduced egg laying in the presence of ethanol within a 60-min time period. These results indicate that SLO-1 channel clusters in egg-laying muscles significantly contribute to ethanol sensitivity (Fig. 6B). Likewise, animals expressing a wild-type copy of the *ctn-1* gene under the control of a pan-neuronal promoter in *ctn-1* mutants, which restores SLO-1 clustering in the neuron (Fig. 2), laid moderately fewer eggs than *ctn-1*(*eg1167*) mutants within a 60-min time period in response to the same ethanol dose. Taken together, these results indicate that, unlike locomotory behavior, ethanol-mediated suppression of egg laying is mediated by clustered SLO-1 channels present in both egg-laying muscles and neurons.

## Discussions

BK channels are known to mediate ethanol sensitivity across a wide variety of species, including *C*. *elegans*. Here, we demonstrate in a *C*. *elegans* genetic study that the behavioral effects of ethanol are mediated by clustered, but not dispersed, SLO-1 channels. We show that *ctn-1* mutation causes a drastic elimination of clustered SLO-1 channels and, as a consequence, an increase in diffuse SLO-1 channels without affecting their overall level. We found that this *ctn-1* mutation confers ethanol-resistant locomotory and egg-laying behaviors equivalent to *slo-1* null mutations, indicating that the behavioral effect of ethanol is mediated by clustered SLO-1 channels.

Our study provides evidence that some of the functions of SLO-1, such as alcohol intoxication, are mediated uniquely by clustered SLO-1 channels, while other functions, such as asymmetry of olfactory neurons, are mediated by diffuse, scattered SLO-1 channels. Our previous study showed that *ctn-1* mutation abolishes SLO-1 puncta in neurons and muscles, although we were not able to definitively conclude whether *ctn-1* mutation causes any change in diffuse, scattered SLO-1 channels^13,15^. Using a SLO-1::GFP genome-edited strain we now show that *ctn-1* mutation disperses SLO-1 channels without affecting their overall abundance. This steady level of SLO-1 channels in the *ctn-1* mutant is in contrast with the dramatically reduced SLO-1 channel level observed in the *erg-28* mutant. Mutation in the *erg-28* gene causes ineffective exit of SLO-1 channels from the ER, thus resulting in the degradation of a majority of SLO-1 channels and a consequent reduction in SLO-1 channel density^17^. Mutation in the *ctn-1* gene does not suppress the defect in the left-right asymmetry of AWC olfactory neurons observed in *slo-1* gain-of-function mutants, while *erg-28* mutation does^17^. These results indicate that the left-right asymmetry of AWC olfactory neurons is associated with the total number of SLO-1 channels, but not the clustering of SLO-1 channels. On the other hand, *ctn-1* mutants exhibit prominent ethanol-resistant locomotory and egg-laying behaviors, whereas *erg-28* mutants show moderate ethanol-resistant locomotory and weak, if any, egg-laying behaviors.

Another interesting result obtained in our study is how the density of BK channels is regulated. The density of ion channels is often regulated by the retrieval from the plasma membrane and subsequent degradation by proteasomes or endocytosis/lysosomes^20–22^. It is conceivable that SLO-1 channels similarly undergo degradation via proteasome- or endocytosis/lysosome-mediated degradation when they cannot form clusters at presynaptic terminals and presumed muscle excitation sites. Contrary to this belief, our data indicate that the density of SLO-1 channels is not regulated by the retrieval from the plasma membrane. *ctn-1* mutation abolishes SLO-1 channel clustering and causes SLO-1 channels to disperse through neurons and muscles but does not influence the overall levels of SLO-1 channels. Furthermore, because *ctn-1* mutation does not suppress the defect in the left-right asymmetry of *slo-1*(*gf*) AWC neurons, disperse SLO-1 channels are functional and are likely present at the plasma membrane. By contrast, *erg-28* mutation, which disrupts normal SLO-1 channel trafficking in the ER, reduces the abundance of SLO-1 channels by over 70%. Therefore, the major regulatory point of the overall SLO-1 channel level is channel trafficking in the ER.

Why does SLO-1 channel-mediated alcohol intoxication require clustering at presynaptic terminals and the sarcolemma? One possibility is that ethanol can activate both clustered and diffuse SLO-1 channels but only clustered SLO-1 channels are relevant to ethanol sensitivity due to their coupling with calcium channels that mediate behavioral sensitivity to ethanol. It has been reported that BK channels are functionally coupled with a wide variety of calcium channels^23–27^, which localize to distinct subcellular compartments, including pre- and post-synaptic sites, the ER, and muscle excitation sites^28,29^. Although clustered and diffuse SLO-1 channels both increase potassium efflux in response to ethanol, only clustered SLO-1 channels lead to an inhibition of behaviorally-relevant calcium channels, thereby causing sedation at the behavioral level. Alternatively, it is possible that, due to their unique locations, SLO-1 channels localized in the clusters bind to ethanol more readily than diffuse channels do. These clustered SLO-1 channels are found at the major entry sites for calcium ions, such as presynaptic terminals and muscle excitation sites, and thus could be exposed to a specific level of calcium ions that makes channel conformation most favorable for ethanol binding. BK channels have indeed been described to adopt multiple complex conformations depending on calcium and voltage^8,30–32^.

Our study demonstrates that the clustering of SLO-1 at presynaptic terminals or presumed muscle excitation sites plays a critical role in mediating ethanol sensitivity. It is generally thought that ethanol-mediated SLO-1 activation reduces overall neural excitability by hyperpolarizing the entire neuron. However, our study indicates that the depressive behavioral effects of ethanol on both locomotory and egg-laying behavior are mediated by clustered SLO-1 channels, suggesting that ethanol inhibits specific molecular steps through a local hyperpolarization mediated by SLO-1 channels. Given that BK channels are functionally coupled with calcium channels in muscles and neurons, these molecular steps likely involve the functions of calcium channels. While our recent study showed that neuron-specific P/Q-type voltage-gated UNC-2 calcium channels, which localize to presynaptic terminals^33^, indirectly contribute to SLO-1 localization in select cholinergic neurons^15^, whether alcohol-mediated SLO-1 activation suppresses UNC-2 function is not clear. Furthermore, it is not clear which calcium channel is the target of alcohol-mediated SLO-1 activation in egg-laying muscles. In the future, it will be necessary to identify calcium channels that couple with clustered SLO-1 channels and mediate the behavioral effects of ethanol.

Drug targeting of BK channels has been suggested as a tool to control alcohol dependence or alcohol withdrawal^34–37^. Given that both diffuse and clustered BK channels serve overlapping and distinct functions in *C*. *elegans* and mammals^38,39^, targeting clustered BK channels may offer a better therapeutic strategy than indiscriminate drug targeting of BK channels. Blocking BK channel clustering will be an effective way of de-coupling BK channels from calcium channels that mediate behavioral alcohol response while sparing the functions dependent on diffuse, disperse BK channels.

## Methods

### Worm strains and maintenance

All *C*. *elegans* strains were cultured at 20 °C on NGM (nematode growth medium) plates seeded with *E*. *coli* OP50. Table 1 lists all strains along with their genotypes and origins. Transgenic strains were constructed using standard DNA microinjection methods with either the coelomocyte *unc-122p*::GFP(or mCherry) or AIY (Amphid Interneuron Y) neuron *ttx-3p*::RFP co-injection marker^40^.

**Table 1:** List of *C*. *elegans* strains used in this study.

Strain	Genotype	Source	Notes
N2	+	CGC	
HKK94	*ctn-1*(*eg1167*) *I*	Abraham *et al*.^13^	
HKK538	*erg-28*(*gk697770*)	Oh *et al*.^17^	
BZ142	*slo-1*(*eg142lf*)	Davies *et al*.^4^	*slo-1* loss-of-function
HKK796	*slo-1*(*cim105[slo-1::GFP]*)	Oh *et al*.^17^	CRISPR/Cas9, C-terminal GFP fusion to SLO-1
HKK123	*ctn-1*(*eg1167*) *I; cimEx6[myo-3p::GFP*, *myo-3p::ctn-1*, *unc-122p::GFP]*	Abraham *et al*.^13^	*ctn-1* muscle rescue
HKK124	*ctn-1*(*eg1167*) *I; cimEx7[H20p::GFP*, *H20p::ctn-1*, *unc-122p::GFP]*	Abraham *et al*.^13^	*ctn-1* neuronal rescue
HKK838	*erg-28*(*gk697770*) *slo-1*(*cim105*)*V*	Oh *et al*.^17^	
HKK839	*ctn-1*(*eg1167*)*I; slo-1*(*cim105*)*V*	This study	
HKK1039	*ctn-1*(*eg1167*)*I;cimEx100[odr-1p::slo-1::gfp*, *unc-122p::mCherry]*	This study	SLO-1::GFP expression in AWC and AWB (Amphid Wing B cell) neurons
HKK1037	*cimEx98[odr-1p::slo-1::gfp*, *unc-122p::mCherry]*	This study	SLO-1::GFP expression in AWC and AWB neurons
HKK823	*ctn-1*(*eg1167*)*I; slo-1*(*cim105*)*V; cimIs14[unc-129p::mCherry::rab-3*, *odr-1p::GFP]; cimEx70[rgef-1p::ctn-1*,*ttx-3p::RFP]*	This study	neuronal expression of *ctn-1*
HKK821	*ctn-1*(*eg1167*)*I; slo-1*(*cim105*)*V; cimIs14; cimEx69[myo-3p::ctn-1*,*ttx-3p::RFP]*	This study	muscle expression of *ctn-1*

### Ethanol-resistant locomotory behavior assay

All animals used for locomotory assays were day 1 adults (20–24 hours post L4). The overall method for the assay has been previously described^4,41^. The NGM agar plates used for all of the experiments were dried without lids for 25 min in a 60 °C incubator. This drying process was necessary to prevent the animals from exhibiting a swimming-like movement on wet plates, and this process usually reduces the agar to 85–90% of its original volume. After the plates were cooled down to room temperature, a desired amount of ethanol (400 mM), which gives internal ethanol concentration of approximately 50 mM^42^, was applied to the NGM plates. The plates were immediately sealed with parafilm and incubated for an additional 2 hours to equilibrate with the ethanol. Immediately before the assays, three flame-heated copper rings were embedded in the agar surface of the NGM plates. These copper rings allow for the simultaneous comparison of three different genotypes in the same assay plates. We included wild-type and *slo-1*(*lf*) mutant (or *ctn-1* mutant) animals in the ethanol assays as positive and negative controls. Ten animals with a given genotype were incubated for 10 min in unseeded plates to remove attached bacteria and then transferred inside a copper ring. After 10 min of exposure to 0 mM and 400 mM ethanol, video frames from three different genotypes were simultaneously acquired from a dissecting microscope fitted with GO-3 camera (Qimaging) with 500 ms intervals for 2 min. For each genotype, the experiments on ethanol plates were repeated three times. We calculated the average speed of the tested animals using the Tracking Objects option in Image-Pro Plus 7.0 (Media Cybernetics, RRID:SCR_016879). The software algorithm automatically generates new tracks when the paths of animals overlap due to collision.

### Ethanol-resistant egg-laying behavior assay

All animals used for the egg-laying assays were day 1 adults (30 hours post L4). Unlike the locomotory assays, the egg-laying assay requires bacterial food^4,7^. NGM agar plates were dried without lids for 2 hours in a 37 °C incubator and then seeded in a triradiate shape with *E*. *coli* OP50. The next day, three small circular agar plugs (1 cm diameter) were punched out from the outside of the bacterial lawn and ethanol (500 mM) was then added to the holes. The agar plugs were placed back into the holes after the ethanol was halfway absorbed into the agar plate. During this process, we ensured that the ethanol did not come into direct contact with the bacterial food. After being sealed with parafilm, the plates were equilibrated at room temperature for approximately 2 hours. As in the locomotory assay, three copper rings were embedded on the agar surface, but the rings were placed where the bacterial food was spread. These copper rings allow the simultaneous comparison of three different genotypes in the same assay plates. Ten animals were directly transferred to each copper ring, and the number of eggs laid was counted at 60 min. The assay included wild-type and *slo-1*(*lf*) mutant animals in the ethanol assays as negative and positive controls, respectively. The experiments were repeated three times for each genotype.

### Microscopy and time-lapse imaging

Images were acquired using a 63x/1.4 numerical aperture on a Zeiss microscope (Axio-Observer Z1) equipped with Spectra X light engine (Lumencor). Images were captured with an 82% quantum efficiency Zyla 4.2 PLUS sCMOS camera (Andor) using MetaMorph 7.8 (RRID:SCR_002368) at 200 ms intervals with 350 ms exposure time. To quantify the intensity of SLO-1 channel clusters, a 150-pixel line was drawn on the linearly aligned fluorescence clusters in head and body-wall muscles, and its pixel intensity was quantified using a line-scanning method in MetaMorph software package (RRID:SCR_002368). To accommodate the variability within each muscle, data obtained from three neighboring parallel lines were averaged for a single data point. For the quantification in egg-laying muscle, the number of fluorescence clusters was counted from an area of a single vulval muscle cell, which was not overlapped with body wall muscle cells or the ventral nerve cord.

### Western blotting and quantification

Mixed-stage worms were lysed and solubilized in SDS lysis buffer (2% SDS, 100 mM NaCl, 10% glycerol, and 50 mM Tris-HCl, pH 6.8) with sonication. Worm extracts were separated by SDS-PAGE and transferred onto PVDF membranes. The separated proteins were probed with anti-GFP (Cell Signaling Technology, #2956, RRID: AB_1196615) or anti-tubulin (Developmental Studies Hybridoma Bank, AA4.3, RRID:AB_579793) antibodies. The pixel intensities of GFP and tubulin bands were quantified using Adobe Photoshop CC 2015.

### Experimental design and statistical analysis

The speed data (Figs 1A and 4E) and egg-laying data (Figs 5 and 6B) were analyzed using one-way ANOVA with Tukey’s post hoc analysis, and the number of fluorescence clusters was analyzed using a two-tailed t-test (GraphPad Prism 7, RRID:SCR_002798). Statistical analyses for each experiment are detailed in the figure legends.

## Supplementary information

<Emphasis Type="Italic">ctn-1(eg1167)</Emphasis> mutant animals exhibit ethanol-resistant locomotory behavior comparable to that of <Emphasis Type="Italic">slo-1(eg142)</Emphasis> mutant animals.

Low-level fluorescent SLO-1 signals in the dorsal cord of <Emphasis Type="Italic">ctn-1(eg1167)</Emphasis> mutant animals are highly mobile, while clustered SLO-1 channels in the dorsal cord of wild-type animals are stable.

BK channel clustering is required for normal behavioral alcohol sensitivity in <Emphasis Type="Italic">C. elegans</Emphasis>

## References

[1] Bettinger JC, Davies AG (2014). The role of the BK channel in ethanol response behaviors: evidence from model organism and human studies. Frontiers in physiology.

[2] Dopico AM, Bukiya AN, Martin GE (2014). Ethanol modulation of mammalian BK channels in excitable tissues: molecular targets and their possible contribution to alcohol-induced altered behavior. Frontiers in physiology.

[3] Dopico AM, Bukiya AN, Kuntamallappanavar G, Liu J (2016). Modulation of BK Channels by Ethanol. International review of neurobiology.

[4] Davies AG (2003). A central role of the BK potassium channel in behavioral responses to ethanol in *C*. *elegans*. Cell.

[5] Mulholland PJ (2009). Sizing up ethanol-induced plasticity: the role of small and large conductance calcium-activated potassium channels. Alcoholism, clinical and experimental research.

[6] Bukiya AN (2014). An alcohol-sensing site in the calcium- and voltage-gated, large conductance potassium (BK) channel. Proceedings of National Academy of Science USA.

[7] Davis SJ, Scott LL, Hu K, Pierce-Shimomura JT (2014). Conserved single residue in the BK potassium channel required for activation by alcohol and intoxication in C. elegans. J Neurosci.

[8] Liu J, Vaithianathan T, Manivannan K, Parrill A, Dopico AM (2008). Ethanol modulates BKCa channels by acting as an adjuvant of calcium. Molecular Pharmacology.

[9] Bukiya AN, Liu J, Dopico AM (2009). The BK channel accessory beta1 subunit determines alcohol-induced cerebrovascular constriction. FEBS Lett.

[10] Kim H (2009). The dystrophin complex controls bk channel localization and muscle activity in *Caenorhabditis elegans*. PLoS Genetics.

[11] Kim H, Oh KH (2016). Protein Network Interacting with BK Channels. International review of neurobiology.

[12] Janssens B, Staes K, van Roy F (1999). Human alpha-catulin, a novel alpha-catenin-like molecule with conserved genomic structure, but deviating alternative splicing. Biochim Biophys Acta.

[13] Abraham LS, Oh HJ, Sancar F, Richmond JE, Kim H (2010). An alpha-catulin homologue controls neuromuscular function through localization of the dystrophin complex and BK channels in *Caenorhabditis elegans*. PLoS Genetics.

[14] Oh HJ (2012). Interaction of alpha-catulin with dystrobrevin contributes to integrity of dystrophin complex in muscle. Journal of Biological Chemistry.

[15] Oh KH (2015). Presynaptic BK channel localization is dependent on the hierarchical organization of alpha-catulin and dystrobrevin and fine-tuned by CaV2 calcium channels. BMC neuroscience.

[16] Chen B (2010). alpha-Catulin CTN-1 is required for BK channel subcellular localization in *C*. *elegans* body-wall muscle cells. EMBO J.

[17] Oh KH (2017). ERG-28 controls BK channel trafficking in the ER to regulate synaptic function and alcohol response in C. elegans. eLife.

[18] Alqadah A (2016). SLO BK Potassium Channels Couple Gap Junctions to Inhibition of Calcium Signaling in Olfactory Neuron Diversification. PLoS Genetics.

[19] White JG, Southgate E, Thomson JN, Brenner S (1986). The structure of the nervous system of the nematode *Caenorhabditis elegans*. Philos Trans R Soc Lond B Biol Sci.

[20] Manna PT (2010). Constitutive endocytic recycling and protein kinase C-mediated lysosomal degradation control K(ATP) channel surface density. Journal of Biological Chemistry.

[21] Felix R, Weiss N (2017). Ubiquitination and proteasome-mediated degradation of voltage-gated Ca2+ channels and potential pathophysiological implications. Gen Physiol Biophys.

[22] Conrad R (2018). Rapid Turnover of the Cardiac L-Type CaV1.2 Channel by Endocytic Recycling Regulates Its Cell Surface Availability. iScience.

[23] Berkefeld H (2006). BKCa-Cav channel complexes mediate rapid and localized Ca2+- activated K+ signaling. Science.

[24] Isaacson JS, Murphy GJ (2001). Glutamate-mediated extrasynaptic inhibition: direct coupling of NMDA receptors to Ca(2+)-activated K+ channels. Neuron.

[25] Wellman GC, Nelson MT (2003). Signaling between SR and plasmalemma in smooth muscle: sparks and the activation of Ca2+ -sensitive ion channels. Cell Calcium.

[26] Weaver AK, Olsen ML, McFerrin MB, Sontheimer H (2007). BK channels are linked to inositol 1,4,5-triphosphate receptors via lipid rafts: a novel mechanism for coupling [Ca(2+)](i) to ion channel activation. Journal of Biological Chemistry.

[27] Zhang J (2018). Glutamate-activated BK channel complexes formed with NMDA receptors. Proceedings of National Academy of Science USA.

[28] Wang X, Wang G, Lemos JR, Treistman SN (1994). Ethanol directly modulates gating of a dihydropyridine-sensitive Ca2+ channel in neurohypophysial terminals. J Neurosci.

[29] Balino P, Ledesma JC, Aragon CM (2014). *In vivo* study of ethanol-activated brain protein kinase A: manipulations of Ca2+ distribution and flux. Alcoholism, clinical and experimental research.

[30] Horrigan FT, Aldrich RW (1999). Allosteric voltage gating of potassium channels II. Mslo channel gating charge movement in the absence of Ca(2+). Journal of General Physiology.

[31] Geng Y, Magleby KL (2014). Single-channel kinetics of BK (Slo1). channels. Frontiers in physiology.

[32] Shelley C, Niu X, Geng Y, Magleby KL (2010). Coupling and cooperativity in voltage activation of a limited-state BK channel gating in saturating Ca2+. Journal of General Physiology.

[33] Saheki Y, Bargmann CI (2009). Presynaptic CaV2 calcium channel traffic requires CALF-1 and the alpha(2)delta subunit UNC-36. Nature neuroscience.

[34] Ghezzi A, Krishnan HR, Atkinson NS (2014). Susceptibility to ethanol withdrawal seizures is produced by BK channel gene expression. Addict Biol.

[35] N’Gouemo P, Morad M (2014). Alcohol withdrawal is associated with a downregulation of large-conductance Ca(2)(+)-activated K(+) channels in rat inferior colliculus neurons. Psychopharmacology.

[36] Kreifeldt M, Le D, Treistman SN, Koob GF, Contet C (2013). BK channel beta1 and beta4 auxiliary subunits exert opposite influences on escalated ethanol drinking in dependent mice. Frontiers in integrative neuroscience.

[37] Scott LL (2018). A Novel Peptide Restricts Ethanol Modulation of the BK Channel *In Vitro* and *In Vivo*. The Journal of pharmacology and experimental therapeutics.

[38] Kaufmann WA (2009). Large-conductance calcium-activated potassium channels in purkinje cell plasma membranes are clustered at sites of hypolemmal microdomains. J Comp Neurol.

[39] Kaufmann WA, Kasugai Y, Ferraguti F, Storm JF (2010). Two distinct pools of large-conductance calcium-activated potassium channels in the somatic plasma membrane of central principal neurons. Neuroscience.

[40] Mello C, Fire A (1995). DNA transformation. Methods in cell biology.

[41] Davies, A. G., Blackwell, G. G., Raabe, R. C. & Bettinger, J. C. An Assay for Measuring the Effects of Ethanol on the Locomotion Speed of Caenorhabditis elegans. *Journal of visualized experiments: JoVE*, 10.3791/52681 (2015). 10.3791/52681 PMC4476067 25938273

[42] Alaimo JT (2012). Ethanol metabolism and osmolarity modify behavioral responses to ethanol in C. elegans. Alcoholism, clinical and experimental research.

