# Supplementary material for: BK channel clustering is required for normal behavioral alcohol sensitivity in C. elegans
Source: Sci Rep. 2019 Jul 15;9:10224. doi: 10.1038/s41598-019-46615-9 (PMC6629859; doi:10.1038/s41598-019-46615-9)
Supplement: Supplementary file 3 — BK channel clustering is required for normal behavioral alcohol sensitivity in C. elegans [file 41598_2019_46615_MOESM3_ESM.pdf]

Supplementary Information

BK channel clustering is required for normal behavioral alcohol sensitivity in *C. elegans*

Kelly H. Oh and Hongkyun Kim

Center for Cancer Cell Biology, Department of Cell Biology & Anatomy, Chicago Medical School, Rosalind Franklin University, North Chicago, Illinois 60064

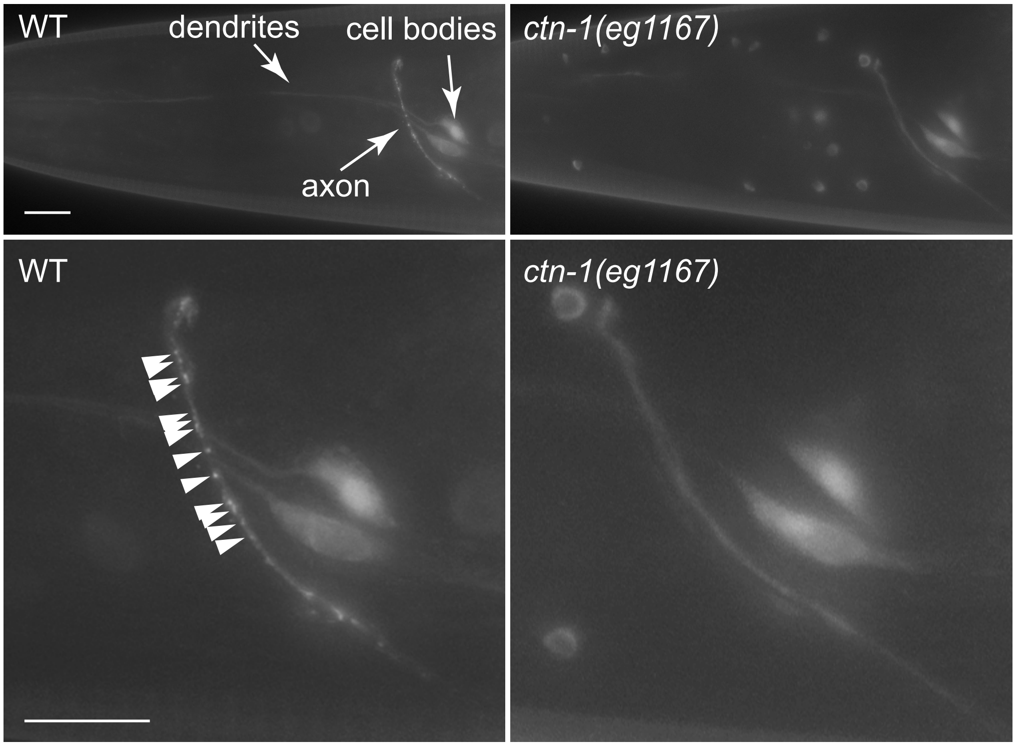

Supplementary Figure 1. *ctn-1* mutation abolishes SLO-1 clustering in AWB and AWC neurons. SLO-1::GFP was expressed under the control of the *odr-1* promoter, which is expressed in AWB and AWC neurons of wild-type (WT) and *ctn-1(eg1167)* mutant animals. White arrowheads indicate SLO-1::GFP puncta in the axons. Scale bar: 10 μm.

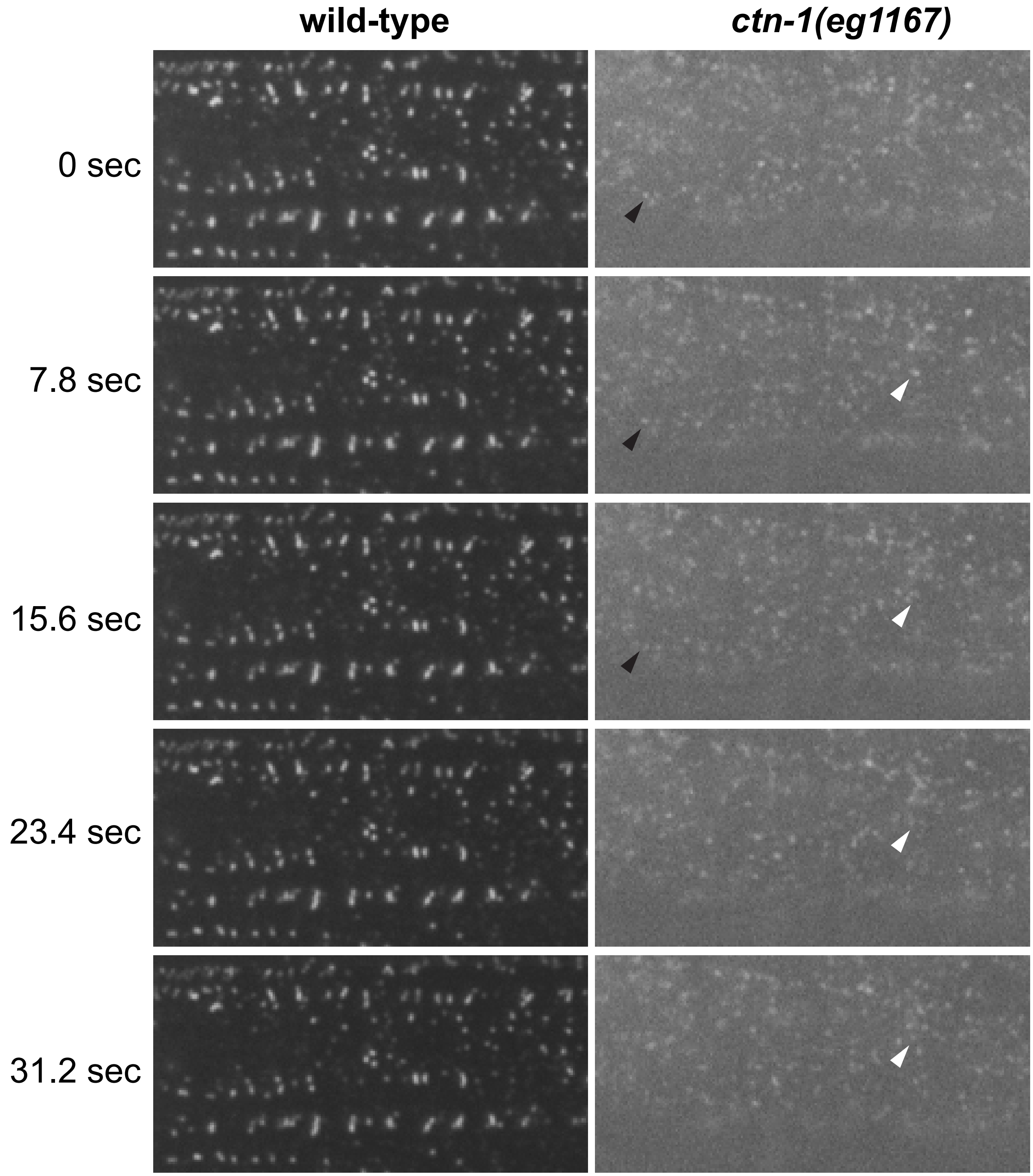

Supplementary Figure 2. Low-level fluorescent SLO-1 signals in the sarcolemma of *ctn-1(eg1167)* mutant animals are highly mobile and fail to form larger stable clusters. Five selected frames of a time-lapse image (200 ms intervals, 350 ms exposure). Wild-type animals show very stable fluorescent SLO-1::GFP clusters in the sarcolemma during the recording period. By contrast, *ctn-1(eg1167)* mutant animals exhibit unstable transient SLO-1::GFP signals in the sarcolemma. The black arrowhead shows an example of a fluorescence signal that disappears and the white arrowhead shows an example of a fluorescence signal that transiently appears.

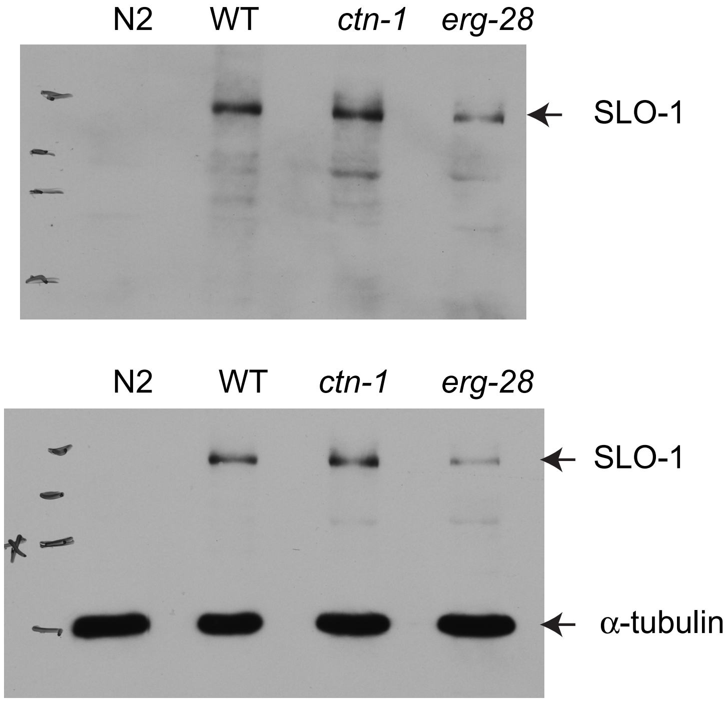

Supplementary Figure 3. Western blot analysis of SLO-1 and alpha-tubulin. Western blot analysis was performed sequentially with anti-GFP (rabbit monoclonal) and anti-tubulin (mouse-monoclonal) antibodies.

Supplementary Video 1. *ctn-1(eg1167)* mutant animals exhibit ethanol-resistant locomotory behavior comparable to that of *slo-1(eg142)* mutant animals. A 2-min video was taken in a 500 ms interval.

Supplementary Video 2. Low-level fluorescent SLO-1 signals in the dorsal cord of *ctn-1(eg1167)* mutant animals are highly mobile, while clustered SLO-1 channels in the dorsal cord of wild-type animals are stable. Fifty frames of a time-lapse image (200 ms intervals, 350 ms exposure).
